# Sex Differences in Rest-Activity Circadian Rhythm in Patients With Metabolic Syndrome

**DOI:** 10.3389/fphys.2021.641461

**Published:** 2021-03-18

**Authors:** Antonino Mulè, Eleonora Bruno, Patrizia Pasanisi, Letizia Galasso, Lucia Castelli, Andrea Caumo, Fabio Esposito, Eliana Roveda, Angela Montaruli

**Affiliations:** ^1^Department of Biomedical Sciences for Health, University of Milan, Milan, Italy; ^2^Fondazione IRCCS Istituto Nazionale dei Tumori di Milano, Milan, Italy; ^3^IRCCS, Istituto Ortopedico Galeazzi, Milan, Italy

**Keywords:** metabolic syndrome, chronobiology, gender differences, circadian rhythms, rest-activity circadian rhythm, intradaily variability, activity level, actigraphy

## Abstract

Rest-Activity circadian Rhythm (RAR) can be used as a marker of the circadian timing system. Recent studies investigated the relationship between irregular circadian rhythms and cardiovascular risk factors such as hypertension, obesity, and dyslipidemia. These factors are related to the Metabolic Syndrome (MS), a clustering of metabolic risk factors that increases the risk of several cardiovascular and metabolic diseases. This cross-sectional analysis aimed to explore the RAR characteristics by actigraphy in subjects with MS, particularly in relation to sex and MS parameters, using parametric and non-parametric analyses. Distinguishing the characteristics of RAR based on sex could prove useful as a tool to improve the daily level of activity and set up customized activity programs based on each person’s circadian activity profile. This study showed that female participants exhibited higher values than male participants in the Midline Estimating Statistic of Rhythm (MESOR) (243.3 ± 20.0 vs 197.6 ± 17.9 activity count), Amplitude (184.5 ± 18.5 vs 144.2 ± 17.2 activity count), which measures half of the extent of the rhythmic variation in a cycle, and the most active 10-h period (M10) (379.08 ± 16.43 vs 295.13 ± 12.88 activity count). All these parameters are indicative of a higher daily activity level in women. Female participants also had lower Intradaily Variability (IV) than male participants (0.75 ± 0.03 vs 0.85 ± 0.03 activity count), which indicates a more stable and less fragmented RAR. These preliminary data provide the first experimental evidence of a difference in RAR parameters between male and female people with MS.

## Introduction

Circadian rhythms influence daily behavior, as well as psychological and physiological functions. Moreover, circadian rhythms can change during the lifespan, both in physiological and pathological conditions (Montaruli et al., 2017). These factors can also be considered markers of a state of health or, otherwise, of disease. For example, the Rest-Activity circadian Rhythm (RAR), which is the expression of 24-h spontaneous activity, can be considered a reliable marker of the circadian timing system. Several studies have investigated RAR alteration, considering the natural aging process and a pathological state (Cornelissen and Otsuka, 2017; Galasso et al., 2020). During aging, RAR displays a decrease in Amplitude, loss of rhythm (Kripke et al., 2005), reduction of adaptability to phase resetting signals (Hofman and Swaab, 2006), and delayed phase (Chaudhury et al., 2005). Alteration in the RAR is also associated with compromised health: psychiatric subjects present decreases of MESOR (Midline Estimating Statistic of Rhythm) and Amplitude (Roveda et al., 2018). Furthermore, people with lower RAR Amplitude are exposed to greater cardiovascular risk (Paudel et al., 2011). Likewise, levels of MESOR and Amplitude were low in people with cancer (Roveda et al., 2019), and some studies have shown that cancer progression tends to be accelerated in patients with alterations of RAR (Mormont and Waterhouse, 2002).

Recent studies have investigated the relationship between irregular circadian rhythms and cardiovascular risk factors such as hypertension, obesity, eating disorders, dyslipidemia, and diabetes (Karatsoreos et al., 2011; Buxton et al., 2012; Galasso et al., 2019).

Metabolic Syndrome (MS) is defined as a cluster of metabolic risk factors such as abdominal obesity, high blood pressure, high fasting glycemia, and dyslipidemia (Alberti et al., 2009), a combination that increases the risk of cardiovascular disease and type 2 diabetes mellitus (Hanson et al., 2002; Mottillo et al., 2010).

Several recent studies have focused on the relationship between sex and MS. Kuk and Ardern (2010) showed more altered values of triglycerides, high-density lipoprotein (HDL), and blood pressure in men than in women. By contrast, Ervin (2009) noted a higher prevalence of abdominal obesity and low HDL cholesterol in women.

Increasing observational and experimental evidence indicates that improving the level of daily physical activity may significantly lower the risk of MS (Buscemi et al., 2014; Miglani et al., 2015), by reducing overweight, waist circumferences, diastolic blood pressure, triglycerides, and fasting plasma glucose (Zhu et al., 2004). It would therefore be useful to investigate the daily activity levels in subjects with MS to develop powerful intervention tools for its treatment, and lifestyle correction.

Up to now, only one study has examined RAR in subjects with MS using actigraphy. Sohail et al. (2015) undertook a non-parametric 7-day analysis of actigraphic data and showed that subjects with more regular RAR had a lower risk of MS or its components and lower risk of cardiovascular disease.

Active people are less likely to develop MS than people with an inactive lifestyle (Zhu et al., 2004; Hajian-Tilaki et al., 2014; Choi and Ainsworth, 2016). However, there have been scant studies of relevant factors such as daily physical activity and the characteristics of RAR related to sex and MS parameters. Several studies have focused on sex differences relating to how high-, moderate-, light-intensity structured physical activity or inactivity affect the risk of MS (Zhu et al., 2004; Choi and Ainsworth, 2016), and report a direct relationship between the level of physical activity and risk of MS. However, it is still not clear which sex is more likely to be exposed to the risk of inactivity and which tends to be more active.

The present study aimed to analyze the RAR characteristics by actigraphy in subjects with MS, particularly in relation to sex and MS parameters, using parametric and non-parametric analyses. The possibility of differentiating the characteristics of RAR based on sex and MS parameters could be useful in improving the levels of daily activity, establishing customized activity programs based on each subject’s circadian activity profile.

## Materials and Methods

### Participants

The present report describes a cross-sectional analysis of baseline data of people with MS from the “Me.Me.Me.” study (ERC-AdG-2012 n.322752), a randomized controlled trial of *Mediterranean diet and Metformin* for the prevention of age-related non-communicable chronic diseases (Pasanisi et al., 2017), developed by Fondazione IRCCS Istituto Nazionale dei Tumori di Milano (Milan, Italy).

The inclusion criteria were:

- presence of MS, based on at least three out of five of the following (Alberti et al., 2009): *fasting plasma glucose* ≥ 100 mg/dL, *blood pressure* ≥ 130/85 mmHg, *high-density lipoprotein* (HDL) < 50 mg/dL for women and < 40 mg/dL for men, *triglycerides* ≥ 150 mg/dL and *waist circumference* of ≥ 100 cm in men and ≥ 85 cm in women (Haftenberger et al., 2002)
- age ≥ 50.

The exclusion criteria were:

- diagnosis of diabetes or fasting glycemia > 126 mg/dL in two consecutive blood samples
- concomitant treatment with Metformin, an insulin sensitizer drug
- serum creatinine > 124 μL/L
- proteinuria
- renal, cardiac, or hepatic insufficiency
- excessive alcohol consumption
- diagnosis of cancer in the last 5 years.

Before beginning the study, all risks and benefits were explained and informed consent was obtained from each participant. We then recorded anthropometric and MS parameters and checked for exclusion criteria. Participants were instructed to wear an actigraph to investigate their activity level, and told how the actigraph unit worked, to ensure accurate actigraphic monitoring.

The study was carried out in accordance with the Declaration of Helsinki and approved by the Ethical Review Board of the National Cancer Institute, Milan.

### Measurements

Participants were examined for:

- anthropometric characteristics (height, weight, BMI, waist circumference); the measurements were made with a body segmental impedance balance, BC- 418 (Tanita); height and weight were measured without shoes and heavy clothes; waist circumference was measured with a tape in the mid-point between the lowest rib and the iliac crest during exhale
- systolic and diastolic blood pressure were measured in triplicate (every 10 min) using an electronic sphygmomanometer (DynaPulse 5000A Pulse Metric, Inc., Vista, CA, United States), with the cuff placed on the left arm and the subject supine, after 10 min of rest
- glycemia (mg/dL), total cholesterol (mg/dL), high density lipoproteins (HDL, mg/dL), low density lipoproteins (LDL, mg/dL), and triglycerides (mg/dL) were assayed in blood samples collected between 8:00 and 9:30, after overnight fasting
- continuous 7-day actigraphic monitoring to detect RAR: the actigraph (MotionWatch 8^®^, CamNtech, Cambridge, United Kingdom) was worn on the non-dominant wrist, and participants were instructed to remove it when swimming or bathing. Subjects received a diary to record bed-time, getting up time, hours of naps, hours without wearing the actigraph, and the number of nocturnal awakenings. Actigraphic monitoring started at the first clinical visit and was completed for all subjects within 30 days. During monitoring, subjects continued their usual activities of teaching, office work, or housework.

### Data Analysis

#### Actigraphic Data

The Actiwatch Software (MotionWatch 8^®^, CamNtech, Cambridge, United Kingdom) was used to obtain the activity levels, expressed in activity counts (a.c.) and recorded every 60 s over the duration of monitoring (7 days). We used a low actigraphic sensitivity threshold (20 counts per epoch).

According to diary entries, subjects removed the actigraph, on average, once a day for about 30 min. To avoid bias, this period was not included in the data analysis. To determine the RAR characteristics, the activity data were analyzed using parametric and non-parametric analysis. The sample was stratified by sex, in male and female groups.

#### Actigraphic Data: Parametric Analysis

The single cosinor method (Halberg et al., 1977; Nelson et al., 1979) describes the time course of activity using an oscillatory function *f*(t) = M + A cos (ωt + φ). This function returns three parameters (M is the MESOR – Midline Estimating Statistic of Rhythm; A is the Amplitude; φ is the Acrophase) that depict a parametric portrait of the activity rhythm for each subject. Briefly, the MESOR is the rhythm-adjusted mean that approximates the arithmetical mean of the data for a given period. The Amplitude measures half the extent of the rhythmic variation in a cycle. The Acrophase indicates the time interval within which the highest activity values are expected. The angular frequency of oscillation is ω and corresponds to the ratio 2π/t, where t is the period of oscillation.

For each participant, we investigated a statistically significant RAR and the three rhythmometric parameters characterizing the rhythm: MESOR, Amplitude, and Acrophase. Then, using the population mean cosinor, we made a rhythmometric analysis to assess the rhythm characteristics of the male and female groups (Nelson et al., 1979). RAR parameters of the two groups were compared with the Hotelling *T*^2^ test, a generalization of Student’s *t*-test used to investigate differences between the means of different populations, or Mann–Whitney tests.

#### Actigraphic Data: Non-Parametric Analysis

The magnitude of stability and fragmentation of RAR for each subject were analyzed with a non-parametric statistical method (Blume et al., 2016) to study non-parametric indices, including Interdaily Stability (IS), Intradaily Variability (IV), the average activity during the least active 5-h period (L5), L5 start time (L5st), the average activity during the most active 10-h period (M10), and M10 start time (M10st). IS indicates the day-to-day variability, providing information on the RAR synchronization with environmental stimuli; it ranges from 0, lack of synchronization, to 1 for perfect synchronization. IV provides information about RAR fragmentation, for example, due to a daytime nap or waking up in the night, which leads to a failure to maintain adequate activity levels over the 24-h profile. It ranges from 0 to 2, with higher values indicating a more fragmented rhythm. L5 indicates the nocturnal activity in people with normal sleep-wake cycles, but in nocturnal workers, it represents the diurnal activity. L5st gives information about the start time of L5 parameters. M10 is the daily activity in people with normal sleep-wake cycles, and in nocturnal workers, it represents the nocturnal activity. M10st gives information about the start time of M10 parameters.

The normality of the data was investigated with the Shapiro-Wilk test and only IS and IV were normally distributed. To compare the two male and female groups, we used a Student *t*-test for IS and IV, and a Mann-Whitney test for L5 and M10.

#### Anthropometric, Cardiovascular, and Metabolic Data

We investigated the differences in age, BMI, waist circumferences, blood pressure, glycemia, HDL, and triglycerides between male and female participants. The Shapiro-Wilk test was used to check the normality of the data. In the total sample age, weight, and waist circumferences were not normally distributed, while blood pressure, glycemia, HDL, and triglycerides were normally distributed. For the male and female group, age, height, blood pressure, and glycemia were normally distributed, while weight, waist circumference, HDL, triglycerides, and BMI parameters were not normally distributed.

We used the Student-*t* or Mann-Whitney tests to compare the anthropometric, cardiovascular, and metabolic characteristics of the two groups. Multivariate correlation analysis was done between anthropometric-, MS- and RAR parameters. This approach was used both for the total sample and for the male and female groups.

## Results

The total sample consisted of 100 subjects, most of them retired; the others were office employees or day workers. In our cohort only one man worked a nightshift and was excluded from the analysis. Two women, and the night shift worker, did not participate in actigraphic monitoring. Data on 97 subjects were analyzed, including 50 female (62 ± 6.2 years) and 47 male (64 ± 6.0 yrs) participants. The proportion of missing data (showering or other activity that could have affected the actigraph) for the 7-day monitoring in the total sample averaged 3.6% (2.4% for male and 4.8% for female). The mean period of missing data did not differ between male and female groups. Table 1 summarizes the anthropometric and metabolic characteristics for the total sample and by sex.

**TABLE 1:** Anthropometric and metabolic syndrome characteristics of the total sample, females, and males groups.

Parameters	Total sample (*n* = 97)	Females (*n* = 50)	Males (*n* = 47)
Age (yrs)	63 ± 6.2	62 ± 6.2	64 ± 6.0
BMI (Kg/m^2^)	32 ± 5.1	32 ± 5.4	32 ± 4.7
Weight (Kg)	89 ± 17.2	83 ± 15.3*	96 ± 16.6*
Height (cm)	166 ± 9.4	160 ± 7.2**	172 ± 6.9**
Waist circumference (cm)	103 ± 12.3	98 ± 11.2°	109 ± 11.1°
Blood pressure (mmHg)	148/89 ± 16.8/9.6	146/88 ± 16.7/8.7	151/90 ± 16.8/10.5
Glycemia (mg/dL)	103 ± 10.3	100 ± 9.4**^§^**	106 ± 10.4**^§^**
HDL (mg/dL)	54 ± 15.4	57 ± 16.8	52 ± 13.3
Triglycerides (mg/dL)	125 ± 53.0	124 ± 51.5	125 ± 55.1
3 MS alterated parameters (%)	45	48	42
4 MS alterated parameters (%)	43	40	45
5 MS alterated parameters (%)	12	12	13

The values are reported as mean ± standard deviation for age, BMI, weight, height, waist circumference, blood pressure, glycemia, HDL, and triglycerides, and as percentages for the MS altered parameters. *, °Mann–Whitney test comparison (*p* < 0.0001); **, ^§^Student *t*-test comparison (respectively, *p* < 0.0001 and *p* = 0.002).

**FIGURE 1:**
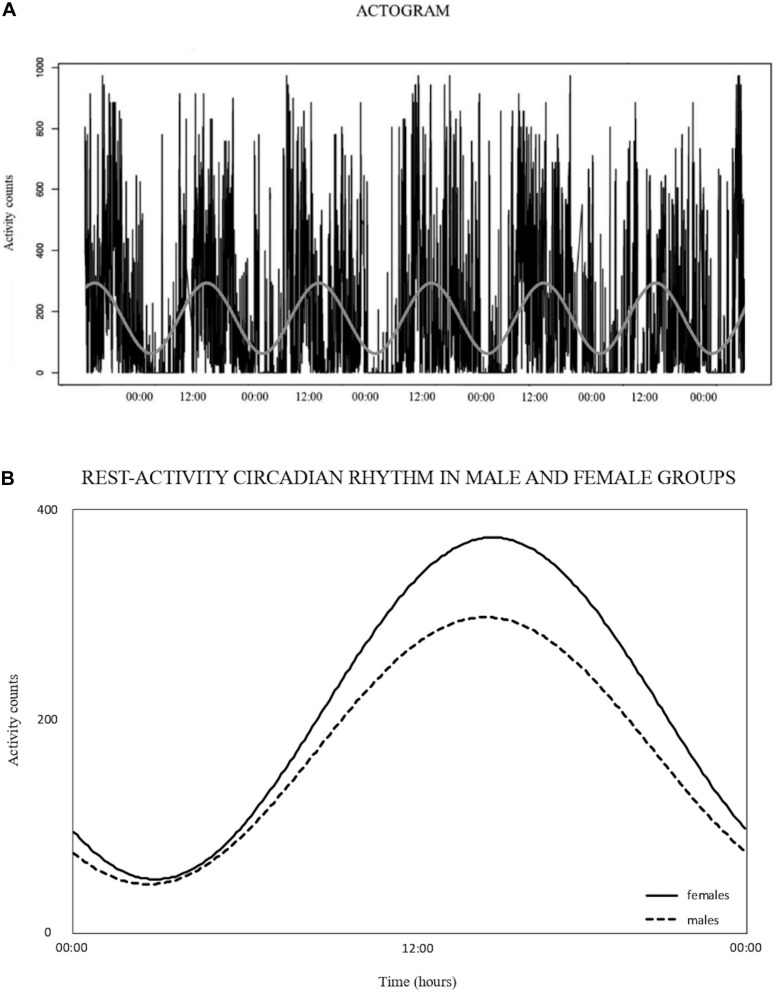
**(A)** Example of actogram expressed in activity counts, collected by a 7-day monitoring. The rest-activity circadian rhythm is represented by the sinusoidal wave resulting from the single cosinor analysis. **(B)** Rest-activity circadian rhythm, expressed in activity counts, resulting from the population mean cosinor analysis for male and female groups. Dashed black line denotes the female group, continued black line denotes the male group.

Male participants had higher values than the female group for weight, height, waist circumferences (*p* < 0.0001), and glycemia (*p* < 0.002). The population means cosinor gave a statistically significant RAR for males and females. The Hotelling *T*^2^ test indicated significant differences for MESOR and Amplitude between male and female groups. In particular, the female participants had higher MESOR and Amplitude than the male group (MESOR: *p* ≤ 0.001; Amplitude: *p* ≤ 0.002). Acrophase did not show any statistically significant differences between the two groups (Table 2; Figure 1).

**TABLE 2:** Rhythmometric analysis of activity levels.

	**Females (*n* = 50)**	**Males (*n* = 47)**
**Parametric analysis**		
MESOR (a.c.)	243.3 ± 20.0*	197.6 ± 17.9*
Amplitude (a.c.)	184.5 ± 18.5°	144.2 ± 17.2°
Acrophase (hh:mm)	14:55 ± 00:41	14:38 ± 00:44
**Non-parametric analysis**		
IS (a.c.)	0.64 ± 0.02	0.62 ± 0.02
IV (a.c.)	0.75 ± 0.03^§^	0.85 ± 0.03^§^
L5 (a.c.)	19.13 ± 1.54	19.63 ± 1.87
M10 (a.c.)	379.08 ± 16.43**^#^**	295.13 ± 12.88^#^

Comparison of female and male RAR parameters from rhythmometric analysis of activity levels. The values are reported as mean ± standard deviation for MESOR, Amplitude, Acrophase, IS, IV, L5, and M10. a.c., activity counts. hh:mm, hours:minutes. *, °Hotelling *T*^2^ test comparison (respectively, *p* = 0.001 and *p* = 0.002), ^§^Student *t*-test comparison (*p* = 0.026), ^#^Mann–Whitney test comparison (*p* = 0.001).

Non-parametric analysis showed a difference for IV and M10, with the females having lower values than males (IV: *p* = 0.026; M10: *p* = 0.001). IS and L5 did not differ between the two groups (Table 2).

L5st and M10st confirmed that L5 represents the night activity (L5st 01:07:26 ± 01:17:32 hh:mm in females; 00:50:22 ± 00:56:04 hh:mm in males) and M10 the daytime activity (M10st 07:33:44 ± 01:45:24 hh:mm in females; 07:05:42 ± 01:50:28 hh:mm in males).

For the females, multivariate correlation analysis (Table 3) showed an inverse correlation between MESOR and age (*p* = 0.018; *r* = −0.33); Amplitude and BMI (*p* = 0.03; Spearman *p* = −0.30); M10 and age (*p* = 0.04; Spearman *p* = −0.30). Linear regression analysis for females showed an inverse relation between MESOR and age (*p* = 0.02; *F* = 5,968; *R*^2^ = 0.11); Amplitude and BMI (*p* = 0.02; *F* = 5.558; *R*^2^ = 0.10); M10 and age (*p* = 0.03; *F* = 4.754; *R*^2^ = 0.09). Thus, in females, aging is associated with a decrease of daily activity and obesity, affecting the Amplitude of RAR. By contrast, in males, multivariate correlation analysis (Table 3) showed a direct relation between MESOR and HDL (*p* = 0.02; *r* = 0.34); Amplitude and HDL (*p* = 0.04; *r* = 0.30); L5 and glycemia (*p* = 0.05; Spearman *p* = 0.29). We found no significant correlations between the other parameters.

**TABLE 3:** Correlation between RAR and MS parameters.

	**Age**	**Weight**	**BMI**	**Wc**	**Sbp**	**Dbp**	**Gly**	**HDL**	**Trg**
**Females group**									
IS	0.164	0.003	–0.156	–0.014	–0.135	–0.055	0.063	0.001	0.269
IV	0.060	–0.070	0.117	0.059	–0.027	0.040	–0.055	0.022	–0.052
L5	–0.203	0.146	0.198	0.157	0.144	0.162	–0.046	–0.182	0.068
M10	−0.295*	–0.166	–0.248	–0.170	–0.034	–0.041	0.121	–0.048	0.075
MESOR	−0.333*	–0.195	–0.263	–0.158	–0.077	0.004	–0.025	0.014	0.023
Amplitude	–0.126	–0.212	−0.303*	–0.198	–0.127	–0.161	0.131	–0.064	0.048
Acrophase	–0.235	–0.057	–0.063	–0.149	–0.125	–0.225	0.117	0.113	–0.028
**Males group**									
IS	0.193	0.189	0.260	0.182	0.076	–0.009	0.065	0.114	–0.028
IV	–0.035	–0.167	–0.165	–0.189	0.248	0.102	0.226	–0.008	0.012
L5	0.230	0.117	0.190	0.095	–0.033	–0.042	0.288*	0.118	–0.132
M10	0.162	–0.017	–0.028	–0.008	–0.063	–0.045	–0.038	0.087	0.142
MESOR	–0.034	–0.022	0.043	–0.027	0.085	0.059	0.022	0.336*	–0.006
Amplitude	–0.151	0.107	0.197	0.111	0.087	0.001	–0.043	0.301*	0.065
Acrophase	0.007	–0.080	–0.078	–0.049	0.057	0.204	0.009	–0.025	0.232

Coefficients of correlation between RAR and MS parameters in females and males. Wc, waist circumference; Sbp, systolic blood pressure; Dbp, diastolic blood pressure; Gly, glycemia; Trg, triglycerides. *p ≤ 0.05.

For male participants, linear regression showed a positive relation between MESOR and HDL (*p* = 0.02; *F* = 5.733; *R*^2^ = 0.11); Amplitude and HDL (*p* = 0.04; *F* = 4.469; *R*^2^ = 0.09). No statistically significant relations were found between the other parameters. These findings indicate that men with higher daily activity levels had higher levels of HDL.

## Discussion

In this cross-sectional analysis, we monitored RAR by actigraphy in subjects with MS to detect the profiles of daily activity levels. The main finding was that RAR differed between male and female participants. The female group had higher MESOR, Amplitude, and M10, indicating a higher level of daily activity than the male group. Female participants also had lower IV than the male group, indicating a more stable and less fragmented RAR. These results provide the first experimental evidence of a difference in RAR parametric and non-parametric parameters between men and women with MS, particularly referred to MESOR, Amplitude, and IV.

Actigraphy is an objective method used to investigate human activity levels. Several researchers have used it to monitor physical activity (Kim et al., 2011, 2013; Scheers et al., 2013) but none of these have conducted a chronobiological analysis of RAR with both parametric and non-parametric analysis. Only one study (Sohail et al., 2015) described the RAR characteristics in subjects with MS but that study aimed to investigate the association between the irregularity of 24-h activity rhythm and the odds of having MS and its components.

The sex differences detected in RAR parameters, particularly in MESOR and Amplitude, indicate higher levels of daily activity in females, supported by the sex gap in M10. Therefore females were more active during the daytime and this could be protective for health. Some recent studies have suggested that altered RAR is associated with compromised health in the elderly. In particular, people with lower RAR Amplitude have a greater cardiovascular risk (Paudel et al., 2011). Lower levels of MESOR and Amplitude have also been reported in people with cancer (Roveda et al., 2019), and the cancer progression was accelerated in patients with RAR alterations (Mormont and Waterhouse, 2002; Innominato et al., 2009). However, no sex differences in rest-activity rhythm parameters were shown in patients with metastatic colorectal cancer (Lévi et al., 2014).

Male participants had higher IV levels than the female group, indicating fragmented activity rhythm. Since L5, the expression of nocturnal activity during the least active 5-h, was similar for the female and male groups, we can speculate that the high IV in males might be related to a fragmented daily activity, probably reflecting periods of daily inactivity.

Some studies have investigated the relationship between irregular circadian rhythms and cardiovascular risk factors such as hypertension, obesity, diabetes, and dyslipidemia (Karatsoreos et al., 2011; Buxton et al., 2012; Shi et al., 2013; Leproult et al., 2014), which are also involved in the development of MS.

Sohail et al. (2015) examined the activity rhythm by actigraphy, using a non-parametric analysis of activity data, and reported that subjects with a more regular activity rhythm had a lower risk of developing MS or its components, such as hypertension, diabetes, dyslipidemia, and obesity. In our sample, the higher IV value in the male participants may be indicative of a higher risk of MS than women.

Therefore MESOR, Amplitude, M10, and IV are the four RAR parameters that might reflect clinical conditions in men compared to women. A structured program of physical activity could act on these parameters, leading to an improvement in daily activity levels, particularly for men.

Several studies have investigated the prevalence of the MS parameters related to sex and their effects on mortality risk (Ford et al., 2002; Ervin, 2009; Kuk and Ardern, 2010). Miccoli et al. (2004) evaluated the prevalence of MS parameters in a cohort of 2,100 Italian adults. They found no significant differences between male and female participants in BMI and waist circumferences, but men had higher blood pressure, glycemia, and triglycerides. The results of our study are partially in agreement with Miccoli et al. (2004) as we found that the groups did not differ in BMI, but there were statistically significant differences in waist circumferences and glycemia.

Regarding the role of physical activity, some researchers have focused on the relationship between the level of physical activity and MS, and showed that active people, both male and female, were less prone to develop MS than inactive people (Zhu et al., 2004; Kim et al., 2011; Choi and Ainsworth, 2016; Li et al., 2018). However, it is not clear which sex is most at risk, and few studies have compared the physical activity levels of both sexes, especially people with MS.

Our results suggest a correlation between RAR and MS parameters. In particular, in female participants, there was an inverse correlation between BMI and Amplitude, MESOR, M10, and age; while in males a direct correlation was found between MESOR, Amplitude and HDL, and between L5 and glycemia. Possibly, by improving the RAR parameters, i.e., MESOR, Amplitude, and M10, we could achieve good results on weight, BMI, and waist circumference.

Several studies have shown that a healthy lifestyle, in particular, adequate levels of physical activity, as well as healthy eating habits, can reduce the development of MS (Zhu et al., 2004; Buscemi et al., 2014; Hajian-Tilaki et al., 2014; Miglani et al., 2015), by acting on overweight (Zhu et al., 2004), waist circumferences, blood pressure, triglycerides and fasting plasma glucose (Wang et al., 2018). Specifically, subjects who spent more time in light-intensity activity and less time sedentary are less likely to develop MS. More generally, subjects without MS were used to more moderate-vigorous physical activity than those subjects with MS, both male and female (Kim et al., 2013).

Our data may agree with this hypothesis. It showed that male participants had higher levels of some MS parameters than the female group and an overall lower level of daily activity, which was confirmed by MESOR, Amplitude, and M10 values, and also a higher sedentary lifestyle during the day, supported by IV.

Monitoring RAR in people with MS could be useful for assessing daily physical activity to reduce health risks and mortality. Unfortunately, very few studies have examined the relationship between MS, RAR, and sex, as well as RAR fragmentation in people with MS. Future studies are now needed to shed light on these aspects that affect MS.

This cross-sectional analysis study suggests that sex might be a distinguishing factor in RAR parameters in MS, although more complex interactions with other variables may be involved and must be investigated. The strengths of the present study are a well-balanced sex sample and objective RAR monitoring by actigraphy during the participants’ usual daily activities. On the other hand, the limitations are the low sample size and the lack of a healthy control group.

Nevertheless, the findings of RAR sex differences encourage us to plan different physical activity programs for male and female patients as part of an intervention study that we are planning with the same subjects.

## Data Availability Statement

The original contributions presented in the study are included in the article/supplementary material, further inquiries can be directed to the corresponding author/s.

## Ethics Statement

The studies involving human participants were reviewed and approved by Ethical Review Board of the National Cancer Institute, Milan. The patients/participants provided their written informed consent to participate in this study.

## Author Contributions

AMu: conceptualization, investigation, methodology, resources, data curation, writing the original draft, writing, review and editing, and supervision. EB: conceptualization, investigation, formal analysis, data curation, writing the original draft, writing, review and editing, and visualization. PP: conceptualization, methodology, investigation, formal analysis, data curation, writing the original draft, writing, review and editing, and visualization. LG: investigation, resources, writing the original draft, writing, review and editing, and visualization. LC: investigation, resources, writing – original draft, writing – review and editing, and visualization. AC: formal analysis, writing the original draft, writing, and review and editing. FE: conceptualization, methodology, writing the original draft, writing, review and editing, and supervision. ER: conceptualization, methodology, data curation, writing the original draft, writing, and review and editing. AMo: conceptualization, methodology, data curation, writing the original draft, writing, review and editing, and supervision. All authors contributed to the article and approved the submitted version.

## Conflict of Interest

The authors declare that the research was conducted in the absence of any commercial or financial relationships that could be construed as a potential conflict of interest.
